# A method for protein extraction from different subcellular fractions of laticifer latex in *Hevea brasiliensis *compatible with 2-DE and MS

**DOI:** 10.1186/1477-5956-8-35

**Published:** 2010-06-18

**Authors:** Xuchu Wang, Minjing Shi, Xiuli Lu, Ruifeng Ma, Chenggong Wu, Anping Guo, Ming Peng, Weimin Tian

**Affiliations:** 1 Key Laboratory of Tropical Crop Biotechnology, Ministry of Agriculture, Institute of Tropical Biosciences and Biotechnology, Chinese Academy of Tropical Agricultural Sciences, Haikou Hainan, China; 2 Key Laboratory of Rubber Biology, Ministry of Agriculture, Rubber Research Institute, Chinese Academy of Tropical Agricultural Sciences, Danzhou Hainan, China

## Abstract

**Background:**

Proteomic analysis of laticifer latex in *Hevea brasiliensis *has been received more significant attentions. However, the sticky and viscous characteristic of rubber latex as cytoplasm of laticifer cells and the complication of laticifer latex membrane systems has made it challenge to isolate high-quality proteins for 2-DE and MS.

**Results:**

Based on the reported Borax/PVPP/Phenol (BPP) protocol, we developed an efficient method for protein preparation from different latex subcellular fractions and constructed high-resolution reference 2-DE maps. The obtained proteins from both total latex and C-serum fraction with this protocol generate more than one thousand protein spots and several hundreds of protein spots from rubber particles as well as lutoid fraction and its membranes on the CBB stained 2-DE gels. The identification of 13 representative proteins on 2-DE gels by MALDI TOF/TOF MS/MS suggested that this method is compatible with MS.

**Conclusion:**

The proteins extracted by this method are compatible with 2-DE and MS. This protein preparation protocol is expected to be used in future comparative proteomic analysis for natural rubber latex.

## Background

Rubber latex is a specific cytoplasm from laticifer cells located in the secondary phloem of rubber tree (*Hevea brasiliensis *Muell. Arg.) [1]. It is a milky fluid composed of a liquid serum [2]. In fresh latex, natural rubber occupies 20-60% of total weight [1]. After ultracentrifugation, latex is separated into three major phases including a top layer of rubber particles, a clear cytoplasm called C-serum, and a pellet known as lutoids [1-3]. C-serum represents the aqueous phase of the laticiferous cytoplasmic content, and contains about 60% of whole latex proteins [4,5]. The lutoids play pivotal roles in coagulating of latex and resisting for outside invasion [6,7]. There are two kinds of roughly distinguished rubber particles called small rubber particle (SRP) and large rubber particle (LRP). Both the LRP and SRP have homogeneous and spherical rubber cores enclosed by a layer of semi-unit membrane [3,8,9].

Till now, the details of natural rubber biosynthesis mechanism in *H. brasiliensis *are still long-standing puzzles [2]. With the new advances in technologies for proteomics, many researchers pay much attention for using these novel approaches to characterize new proteins and outline the details of rubber biosynthesis [8]. By the mid-1990 s, latex proteins Hev b 9 (enolase) and Hev b 10 (Mn SOD) were identified as allergens using proteomic approach [8]. Using 1-DE and 2-DE, several proteomic studies were performed. Firstly, Martin (1991) found both the pellet fraction and cytoplasm in rubber latex contained large amounts of a relatively few kinds of proteins. On 2-DE gels, only several high abundant protein spots were visualized and identified as chitinases/lysozymes [1]. To search the molecular basis of the tapping panel dryness (TPD) disease, Dian and colleagues found 5 TPD-related proteins in latex [10]. Recently, a few proteomic studies were conducted on rubber particles [11], C-serum [12], lutoids [6,13,14] and seeds [15] from *H. brasiliensis *and other latex-producing plants such as *Calotropis procera *[16], *Chelidonium majus *[17] and *Lactuca sativa *[18].

However, these aforementioned results were somewhat low-quality due to both vertical and horizontal streaks on their 2-DE gels, particularly in high-abundant protein regions. This is probably due to the sticky and viscous characteristic of latex that makes the isolation of qualified proteins for proteomic analysis more difficult. Till now, to our best knowledge, there is no effective method specifically devised for isolating proteins from different rubber latex fractions. Latex is rich of compounds such as salt, minerals, lipids, carbohydrates, and in particular complex membrane systems, which can be co-extracted with proteins and interfere with 2-DE [11]. Particularly, rubber elongation factor (REF) accounts for 10-60% of total latex proteins and can badly affect the separation of latex proteins on 2-DE gel [5,15,19], thus making the application of 2-DE for rubber latex proteome remained a challenging job.

In order to construct reference 2-DE maps for the latex proteome, it is of significant to develop a method for preparing high-quality proteins from different fractions of rubber latex. Based on our Borax/PVPP/Phenol (BPP) method that could efficiently extract proteins from recalcitrant plants [20] and chloroplast [21], we developed a new protein preparation protocol for rubber latex. Here we described the details of this protocol and presented high-resolution 2-DE protein profiles for different fractions of latex from *H. brasiliensis*. The comparison of our results with the former studies and the identification of some rubber latex specific proteins suggested our method could be more efficient for 2-DE and MS in future latex proteomic analysis.

## Results and discussion

### Modifications of BPP protocol for protein extraction from different subcellular fractions of rubber latex

**Figure 1:**
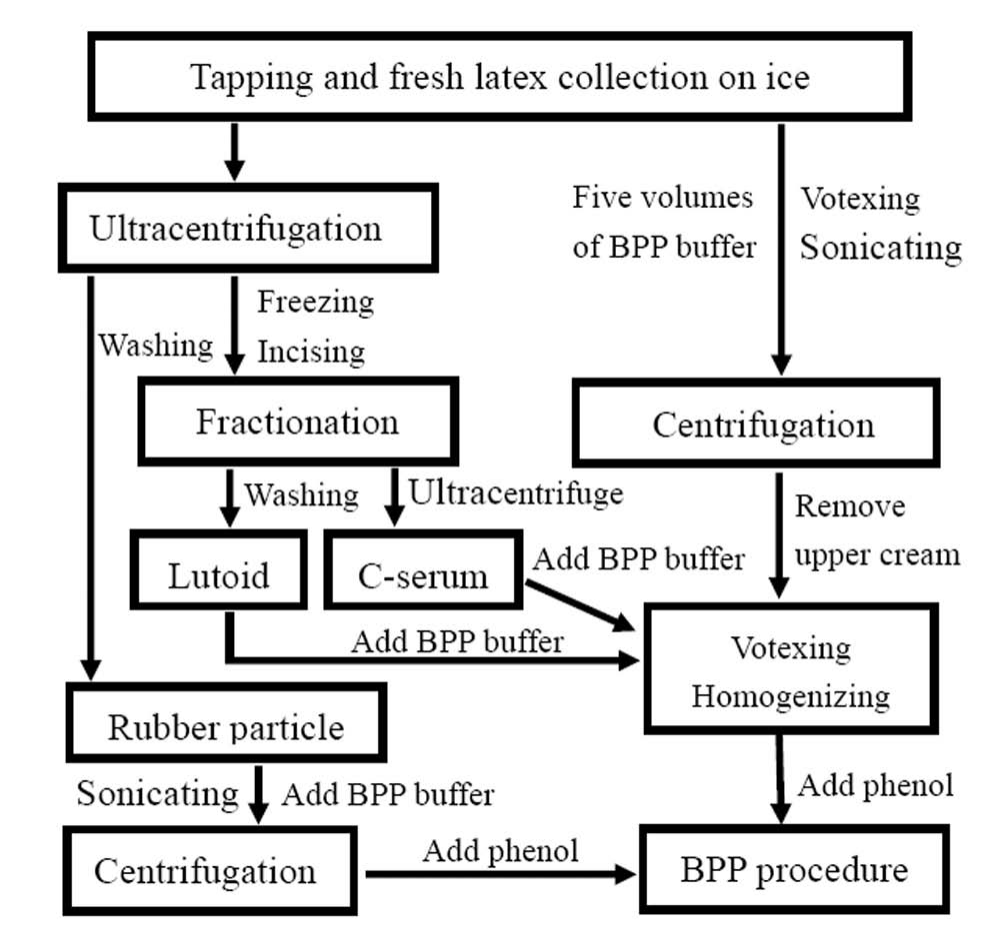
**Schematic outlines of the fractionation-based protein extraction procedure for rubber latex**. The original steps and our improvements are shown in the frames with white background. The details of extraction procedure were described in the materials and methods section.

This protocol was modified from the BPP method to isolate total proteins from halophytes [20], and its modified version for isolation chloroplast proteins [21]. The details of this modified BPP protocol for rubber latex were outlined schematically as below (Fig 1). Firstly, the remained samples were frozen immediately after the first centrifugation, followed by incising into the C-serum and lutoid parts, thus avoiding the contamination of other organic substances. Secondly, purification procedure was used for different latex fractions. The washing steps for crude rubber particles and lutoids got rid of the contaminants such as proteins, lipids, salt ions, polysaccharides, terpenes, carbohydrates, nucleic acids and other organic solutes. The centrifugation of crude C-serum efficiently removed the remained compounds such as lutoids and other organelles.

Thirdly, in this modified BPP version, more protein extraction buffer was used for homogenization (Fig 1). In original BPP protocol, 1 g tissues were mixed with 3 ml BPP buffer [20,22]. However, if this buffer/sample ratio was applied directly to rubber latex, the protein yield reduced sharply for protein losing and insufficient extraction (data not shown). This is probably due to the rubber latex as a cytoplasm from laticifer contains very high concentration of proteins [1,8]. Additionally, the upper layer of rubber cream must be removed after the first centrifugation step for total latex and rubber particle samples. If not, the homogenization would solidify immediately after the addition of Tris-saturated phenol, thus resulting in very low protein yield.

### Modified BPP protocol produced high yield of latex protein

Protein yields produced by this method for total fresh latex, lutoid and C-serum were 7035 ± 314, 5474 ± 219, and 7616 ± 280 μg/ml, respectively (Table 1). The latex known as a specific laticifer fluid is rich of proteins [8,16]. It was reported that total mass of proteins in the whole latex of tropical shrub *Calotropis procera *was about 14.88 mg/ml, and the total soluble proteins among them accounted for 60%, that is, 8.85 mg/ml proteins [16]. In *H. brasiliensis*, the total mass of proteins is about 14.0 mg/ml in total latex if directly determined by Bradford assay [8].

**Table 1:** Protein yields and detected protein spot numbers on 2-DE gels for different rubber latex fractions

Fraction	Protein yield (μg/ml)	SE	Spot number	SE
Total fresh latex	7035	± 314	1358	± 65
Rubber particle	--	--	583	± 25
C-serum	7616	± 280	1248	± 51
Lutoid	5474	± 219	385	± 15
Lutoid membrane	--	--	724	± 33

Note: SE, standard error; --, not detected.

Using modified BPP protocol, we obtained 7.0 mg proteins per milliliter fresh total latex after many extraction steps, accounting for 50% of the total proteins in latex. From purified C-serum, we obtained 7.6 mg/ml proteins (Table 1), which is about 10-fold higher than that provided by Li and coworkers [5]. Using phenol (Phe) extraction method, Li and coworkers obtained only 0.66 mg proteins from 1 ml C-serum. They considered the high clean-up capacity of Phe method might remove some proteins, thus decreasing protein yield. Furthermore, they compared three protein extraction methods for C-serum by protein yield and 2-DE images and found TCA/acetone method generated the highest protein yield (0.86 mg/ml), followed by E-TCA method (0.72 mg/ml) and Phe method [5]. They then concluded Phe method was not suitable for latex C-serum [5].

After checking their extraction procedure carefully, we found there are at least two mistakes in their Phe protocol. One is that they used 0.1 mM not 100 mM ammonium acetate in methanol as protein precipitation reagent. In Phe method, the widely used concentration of ammonium acetate is 100 mM rather than 0.1 mM [20,22,23]. Using five volumes of 0.1 mM ammonium acetate in methanol as protein precipitation reagent, proteins can not be precipitated efficiently and many proteins were lost. Another problem is they used a very simple extraction buffer (100 mM EDTA, 200 mM KCl, 4% β-mercaptoethanol and 2 mM PMSF, 100 mM Tris-HCl, pH 8.5) not including sucrose and other reagents, thus leading to failure of isolation many combining proteins especially membrane proteins.

### Modified BPP method recovered more proteins from different rubber latex fractions on 1-DE and 2-DE gels

**Figure 2:**
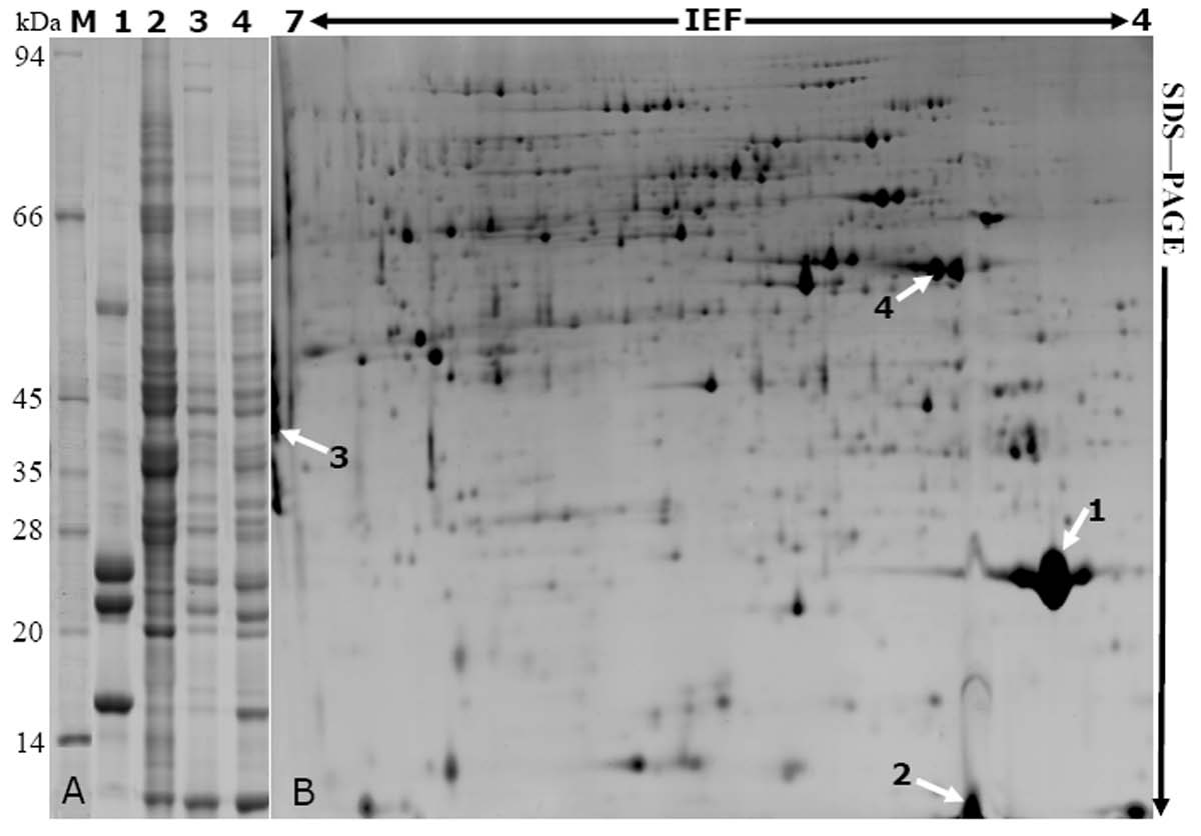
**Protein profiles of rubber latex on both 1-DE and 2-DE gels**. Both 1-DE (A) and 2-DE (B) results of rubber latex proteins from *Hevea brasiliensis *were presented. A, 1-DE analysis of proteins from lutoids (lane 1), C-serum (lane 2), rubber particles (lane 3), and total rubber latex (lane 4), respectively. M represents the molecular weight marker. The molecular masses were indicated on the left. B, 2-DE map of total latex proteins. About 1350 protein spots were detected. The spot number corresponded to that in Table 2.

**Figure 3:**
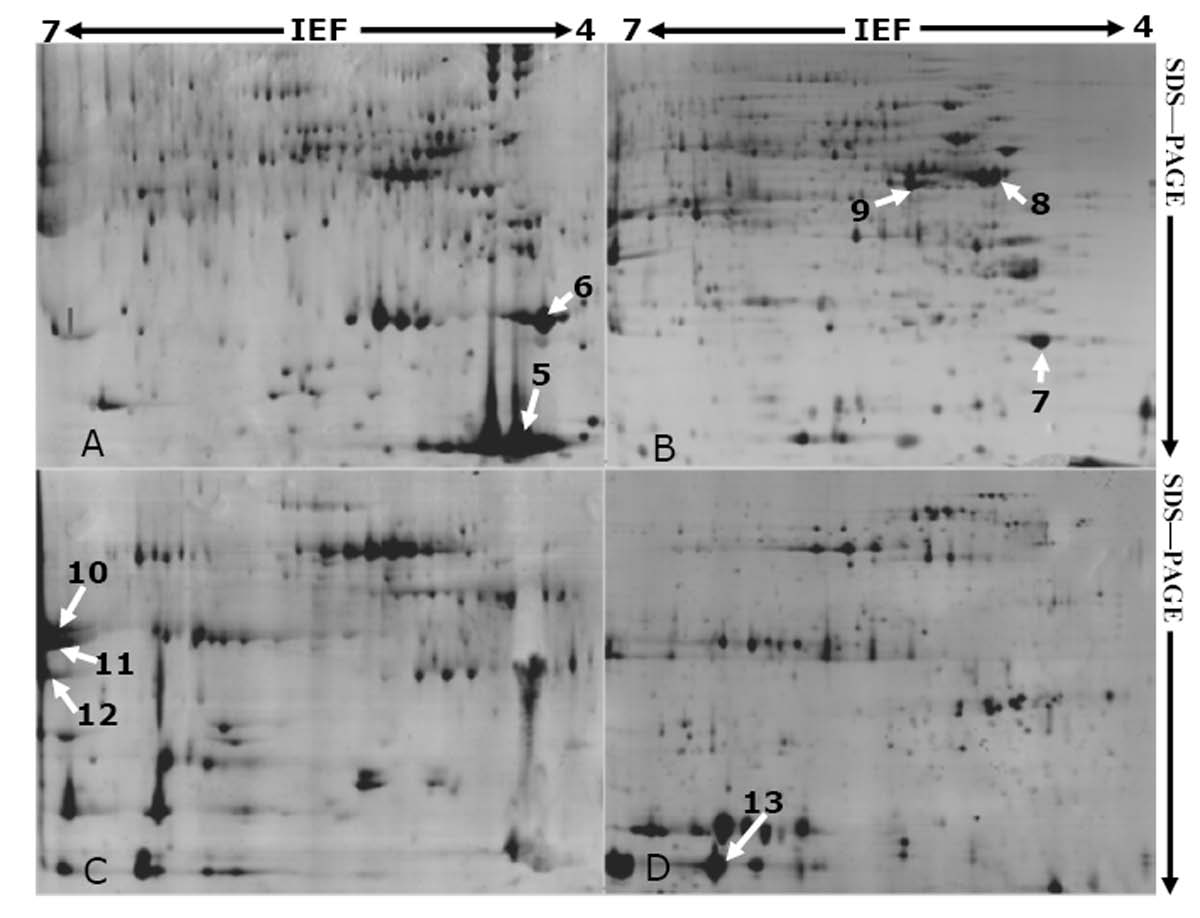
**2-DE profiles of proteins extracted from rubber particles (A), C-serum (B), lutoids (C), and lutoid membrane (D) of natural rubber latex**. About 583, 1248, 385, and 724 protein spots were detected on the 2-DE gels for rubber particles (A), C-serum (B), lutoid (C), and lutoid membrane (D), respectively. The arrows indicate the protein spots with MS identification. The spot number corresponded to that in Table 2.

To determine the protein quality for MS, we further performed 1-DE and 2-DE, and the protein profiles were outlined (Fig. 2; Fig. 3). On 1-DE gel, distinct protein bands were observed at both higher and lower molecular weight (*M*r) regions (Fig. 2A, lanes 1-4). For lutoid sample, only 4 main bands were observed (Fig. 2A, lane 1). This result was consistent with the former reports that only several main bands were observed on 1-DE profiles for lutoids [6,9]. In B-serum, there existed only a smaller number of proteins and fewer than 20 major peptides. Among them, a single protein named hevein making up 50-70% of the total B-serum soluble proteins was identified [8]. However, there were more than 30 distinct protein bands on 1-DE gels of total latex (Fig. 2A, lane 2), rubber particle (Fig. 2A, lane 3), and C-serum fraction (Fig. 2A, lane 4). It was noteworthy that total latex (Fig. 2A, lane 2) and C-serum (Fig. 2A, lane 4) demonstrated similar 1-DE protein profile except for several bands in low *M*r region (Fig. 2A), indicating most varieties of proteins in latex was as same as that in C-serum fraction.

On the 2-DE gels for proteins from total latex (Fig. 2B), rubber particles (Fig. 3A), C-serum (Fig. 3S), lutoids (Fig. 3C) and lutoid membranes (Fig. 3D), the modified BPP protocol generated from several hundreds to more than one thousand of protein spots (Table 1) with a broad distribution at both horizontal and vertical dimensions (Fig. 2B; Fig. 3A-D). The spots showed superior resolution with clear background and minimal streaking and the spot shape appeared round or elliptical, even at both cathode and anode points (Fig. 2B; Fig. 3), or around high abundant protein regions (Fig. 2B, spot 1; Fig. 3A, spots 5 and 6). These results suggested isoelectric focusing (IEF) process was sufficient.

The highest number of protein spots was observed on the total latex 2-DE gel (Table 1). There were 1358 ± 65 visible spots with excellent repetitions on the 2-DE gel of total latex (Fig. 2B). The C-serum fraction demonstrated similar protein profile with total latex sample, which is consistent with the aforementioned 1-DE result (Fig. 2A). More than 1200 visible spots (Table 1) were visualized on the C-serum 2-DE gel (Fig. 3B). From rubber particle fraction, we detected 583 ± 25 protein spots (Table 1; Fig. 3A). The least number (385 ± 15) was generated from the lutoids (Table 1; Fig. 3C), probably due to the existence of several high abundant lutoid proteins as that on 1-DE gel (Fig. 2A, lane 1), which severely limited the amount of target proteins loading onto the IPG strips for IEF and clouded other protein spots, thus resulting in the inaccurate spot positions or failure of detecting some significant proteins.

Although there were several proteomic studies for rubber particles [11], C-serum [12], lutoids [6,13,14] and seeds [15] for *H. brasiliensis *and other latex-producing plants [16-18], most of their 2-DE maps were somewhat low-quality for MS, and there are still large amounts of proteins remained unidentified in different fractions of natural rubber latex presumably due to the low-resolution 2-DE gels as well as the lack of available genome-sequence information.

### Modified BPP method generated high-resolution 2-DE reference maps for lutoids and lutoid membrane proteins

As bottom fraction after ultracentrifugation, lutoids contain mainly specialized vacuoles as well as other organelles such as ribosome and endoplasmic reticulum. These organelles are widely considered to own complicated membrane systems [8]. Although significant advances have been achieved, it is still a challenging work to obtain entire membrane proteins for 2-DE and MS [21]. There has been reported that lower recovery of proteins from *H. brasiliensis *was mainly due to the loss of very hydrophobic proteins such as membrane proteins [15].

Here, we presented a high-resolution 2-DE map of membrane proteins from lutoids (Fig. 3D). There were more visible protein spots for lutoid membrane sample (742 ± 33 spots) than total lutoid fraction (Table 1; Fig. 3C and 3D). After repeated freezing and thawing, about 50-70% of B-serum soluble proteins such as hevein and beta-1,3-glucanase were removed [8,24], so increased many low abundant proteins on the 2-DE gel (Fig. 3C and 3D).

Several groups also tried to resolve the problems in preparation of high-purity latex proteins for 2-DE and MS by evaluating different protein extraction methods. Duan and coworkers isolated the crude extracts of membrane proteins from rubber particles and resolved about 520 spots on the silver stained 2-DE gels, but the 2-DE gels they provided were heavy smearing especially in the high abundant region [11]. In this research, we obtained good-resolution 2-DE map and produced 583 ± 25 well-focused protein spots from rubber particles (Fig. 3A). To isolate total proteins from lutoids, Wei and coworkers evaluated the TCA/acetone, Tris-buffer and phosphoric acid buffer methods, and found Tris-buffer method was a preferred one, which could produce about 300 spots on 2-DE gels [6]. Using modified BPP method, we obtained more protein spots from lutoids than Wei and coworkers. There were 385 ± 15 and 724 ± 33 detectable spots visualized on the gels of total lutoid and lutoid membranes (Table 1; Fig. 3C and 3D).

Using TCA/acetone precipitation method, Yan and Chen extracted lutoid proteins from latex of healthy and TPD trees [13,14]. They performed 2-DE and detected 24 notable changed spots [13], then identified 13 differential expressed proteins by MS [14]. Furthermore, they isolated C-serum proteins from the same plants by TCA/acetone precipitation and obtained about 970 spots on CBB stained 2-DE gels. Finally, they identified 27 proteins in C-serum, some of them were considered to be involved in the onset of TPD syndrome [12]. But their 2-DE results were not quality-enough to perform comparative proteomic analysis due to the limitation of protein extraction method. Finally, they evaluated the E-TCA, TCA/acetone precipitation and Phe methods, and proposed the TCA/acetone method was the most suitable one for protein extracting from the C-serum [5].

In this research, we obtained 1248 ± 51 visible spots for C-serum (Table 1; Fig. 3B). In a recent report, Li and coworkers detected only 447 spots using Phe method [5]. From total fresh latex, we obtained more than 1350 spots on 2-DE gel (Fig. 2B). This value is also far more than that reported by many researchers. Nawrot and colleagues extracted total proteins from the milky sap of Greater Celandine (*Chelidonium majus *L.) and obtained less than 50 protein spots on 2-DE gel stained with silver, they ultimately identified 21 proteins using liquid chromatography-tandem mass spectrometry analysis [17]. Freitas and colleagues performed 2-DE using total laticifer proteins from the latex of *Calotropis procera *and produced protein profile on silver staining 2-DE gels with a very limited number of protein spots [16].

Recently, Cho and coworkers conducted a comprehensive proteome analysis of total latex from lettuce (*Lactuca sativa*) using 1-DE and positively identified 587 latex proteins by multidimensional protein-identification technology [18]. To our best knowledge, this is the first large-scale proteome analysis of lettuce latex till now, and there are indeed many exciting findings. However, compared to our 1-DE results (Fig. 2A, lane 2), the 1-DE gel provided by Cho and coworkers was low-resolution with heavy background and a bit smearing [18], probably due to limit of protein preparation method. They used neither extraction nor purification procedure and only collected the exuding latex and immediately expelled into PBS solution on ice, then directly frozen in liquid nitrogen for further proteomic analysis [18], thus leading to bad separation of proteins.

Using modified BPP method, we obtained high protein yield and excellent 2-DE gels from different fractions of latex probably due to the following reasons: Firstly, we sonicated the total latex, rubber particle and lutoid fraction sufficiently, and then vortexed the mixtures of the sample fractions with the BPP buffer vigorously, thus resulting in the extraction of proteins efficiently. Secondly, we changed the buffer/sample ratio. Thirdly, we used an efficient protein extraction buffer named as BPP buffer. This newly developed buffer contained a nonionic detergent (Triton X-100) and other strong reducing agents such as PVPP, Vitamin C and β-mercaptoethanol, thus making the proteins isolation from the fractions effectively as well as removing the interfering compounds [20]. Finally, we used ammonium sulfate saturated-methanol rather than ammonium acetate saturated-methanol as a protein precipitation reagent as that used in the original BPP method. This new protein precipitation reagent was thought to generate more qualified proteins for 2-DE [20].

### Modified BPP method was compatible with MS

**Figure 4:**
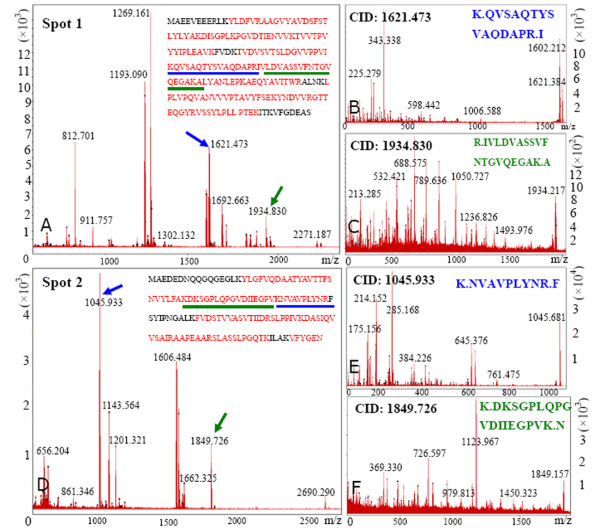
**Identification of proteins from rubber latex *via *MALDI TOF/TOF MS**. The 2 protein spots (spots 1 and 1 from total rubber latex) were excised and digested with trypsin, and then the collected peptides were analyzed using an Autoflex MALDI-TOF/TOF mass spectrometer. The annotated PMF spectral peaks showed the intensities of different peptides (A for spot 1 and D for spot 2). The sequences marked with colour and dark letters represent the matched and unmatched peptides, respectively. The matched peptides marked with under-lines were further identified with MALDI TOF-TOF MS/MS and the annotated spectrum of CID (collision induced dissociation) was presented (B and C from spot 1; E and F from spot 2). Database searching with Mascot software against NCBInr database identified the 2 proteins as SRPP (spot 1) and REF (spot 2) from rubber tree respectively.

To further evaluate the compatibility of this method with MS, we excised some representative protein spots on 2-DE gels for different fractions of rubber latex, which were indicated by arrows and marked with numbers (Fig. 2B; Fig. 3A-D), and then positively identified 13 proteins *via *MALDI TOF MS and were further confirmed by TOF/TOF MS/MS with their identities listing in Table 2 and Suppl Data 1. Among them, 2 proteins from total latex (spots 1 and 2) were picked out to demonstrate their identities in detail (Fig. 4A-F). Their annotated peptide mass fingerprinting (PMF) spectrum (Fig. 4A and 4D), peptide sequences translated from the cDNA sequences (Fig. 4A and 4D), and ions spectrum produced by TOF/TOF (Fig. 4B, C, E, and 4F) for spot 1 (Fig. 4B and 4C) and spot 2 (Fig. 4E and 4F) were demonstrated respectively. Mascot searched results revealed that the 2 proteins were small rubber particle protein (SRPP) (spot 1) and REF (spot 2) respectively (Table 2; Suppl Data 1).

**Table 2:** Proteins identified from rubber latex *via *MALDI TOF/TOF MS/MS

No.^a^	Acc.^b^	Protein name (TOF/TOF sequence)^c^	Exper. *p*I/kDa^d ^	Thero. *p*I/kDa^e^	MP/TP^f^	SC (%)^g^	Scores (PFF)^h^	Plant species
**1**	14423933	Small rubber particle protein (K.QVSAQTYSVAQDAPR.I) (R.IVLDVASSVFNTGVQEGAK.A)	4.71/25	4.80/22	13/62	85 (7) (9)	94 (91) (124)	*H. brasiliensis*
**2**	132270	Rubber elongation factor (K.NVAVPLYNR.F) (K.DKSGPLQPGVDIIEGPVK.N)	4.95/12	5.04/15	10/33	78 (6) (13)	125 (59) (80)	*H. brasiliensis*
**3**	10946499	beta-1,3-glucanase (R.YIAVGNEISPVNR.G)	7.02/34	9.46/35	15/48	47 (3)	103 (50)	*H. brasiliensis*
**4**	3288200	latex allergen (K.SLDCEDYYLR.I) (R.DTYDPIHSIGPIYDGEYLR.E)	4.87/44	5.00/43	22/42	68 (2) (4)	214 (71) (122)	*H. brasiliensis*
**5**	132270	Rubber elongation factor (K.DKSGPLQPGVDIIEGPVK.N)	5.08/15	5.04/15	10/33	60 (13)	126 (76)	*H. brasiliensis*
**6**	14423933	Small rubber particle protein (K.QVSAQTYSVAQDAPR.I)	4.88/24	4.80/22	14/37	87 (7)	161 (91)	*H. brasiliensis*
**7**	14423933	Small rubber particle protein (K.ENENFQQEANEQEEK.L)	4.65/23	4.80/22	13/62	85 (12)	94 (96)	*H. brasiliensis*
**8**	6707018	latex allergen (R.DTYDPIHSIGPIYDGEYLR.E)	5.01/43	5.00/43	15/52	45 (4)	139 (104)	*H. brasiliensis*
**9**	58013197	Actin (K.NYELPDGQVITIGAER.F)	5.12/42	5.31/42	20/60	53 (4)	125 (68)	*I. tinctoria*
**10**	10946499	beta-1,3-glucanase (R.NIHDAIR.S) (R.YIAVGNEISPVNR.G)	6.87/34	9.46/35	20/64	57 (2) (3)	168 (106) (60)	*H. brasiliensis*
**11**	10946499	beta-1,3-glucanase (R.IYDPNQAVLEALR.G)	6.89/33	9.46/35	19/51	68 (3)	173 (107)	*H. brasiliensis*
**12**	157831407	Chain A, Hevamine (K.VYLTAAPQCPFPDR.Y)	7.08/30	8.44/30	7/10	50 (4)	99 (54)	*H. brasiliensis*
**13**	124365253	Chain A, Hydroxynitrile Lyase (K.RPFFTK.E) (K.VTALDLAASGVDPR.Q)	6.22/11	5.15/30	9/24	42 (2) (5)	93 (92) (86)	*H. brasiliensis*

^a^Assigned spot number as indicated in Fig 2 and Fig 3.

^b^Database accession numbers according to NCBInr.

^c^The name of proteins identified by MALDI TOF MS, and the peptide sequences produced by TOF/TOF MS/MS were presented in the bracket.

^d, e^Experimental (d) and theoretical (e) mass (kDa) and p*I *of the identified proteins.

^f^Number of matched peptides with PMF data and the total searched peptides.

^g^The amino acid sequence coverage for the identified proteins.

^h^Mascot score against the database NCBInr for PMF and PFF (in the bracket).

As seen in Fig. 4, the peaks corresponded to peptide isotopic mass clusters had low noise background values (Fig. 4A-F), illustrating both the PMF and peptide fragmentation fingerprinting (PFF) spectra were high-quality and the peptidic spectral artifacts were little. Among the identified 13 ones, 2 proteins (spots 2 and 5) were REF (Table 2) from rubber particle (Fig. 3A) and total latex (Fig. 2B). There were 3 protein spots (spots 1, 6, and 7) identified as SRPP (Table 2; Suppl Data 1) from the total latex (Fig. 2B), rubber particle (Fig. 3A), and C-serum fraction (Fig. 3B). Three protein spots (spots 3, 10, and 11) were identified as beta-1,3-glucanase (Table 2) from the total latex (Fig. 2B) and lutoid fraction (Fig. 3C). Many other typical proteins in rubber latex were also positively identified (Table 2). They were latex allergen (spots 4 and 8), chain A for crystal structures of hevamine (spot 12), chain A for hydroxynitrile lyase (spot 13), and actin (spot 9) (Table 2; Suppl Data 1). These aforementioned results suggested that the modified BPP method is compatible with MS and can be used for further proteomic analysis of rubber latex proteins.

## Conclusions

In this research, we established a modified BPP protocol to extract proteins from different rubber latex fractions. The obtained proteins from both total latex and C-serum fraction generate more than one thousand spots and several hundreds of protein spots from rubber particles as well as lutoid fraction. The identification of 13 rubber latex proteins revealed that this method was compatible with 2-DE and MS. To our best knowledge, it is the first time that a specific method for protein extracting from latex was presented. This protocol is expected to be used in future comparative proteomic analysis for rubber latex.

## Materials and methods

### Plant material

The 8-year-old virgin rubber trees (*H. Brasiliensis *Mull. Arg., clone RY 7-33-97) grown in the experimental farm of the Chinese Academy of Tropical Agriculture Sciences with homogeneous stem girth (53 ± 2 cm) were selected. After tapping, the first 20 drops of latex were discarded and the subsequent latex samples were collected.

### Isolation of different fractions from rubber latex

After the fresh latex was ultracentrifuged (Beckman Model JA 2-21, Beckman Coulter, Fullerton, CA) at 4°C in an angled rotor for 30 min with 40,000 g, 3 major layers were observed. The top white zone was rubber particles, the middle aqueous layer was C-serum, and the bottom fraction was lutoids [24]. After removing the upper cream of rubber particles, the remained samples were immediately put into liquid nitrogen, and the freezing icicles were pulled out and then incised into the C-serum and lutoids parts by cutting with a clean blade carefully. The collected crude fractions of rubber particles, C-serum and lutoids were put into different new tubes respectively.

The collected top creamy rubber particles were resuspended in ice-cold washing solution (WS) (20 mM Tris-HCl, 300 mM mannitol, 0.5 mM DTT, pH 7.2) in ration of 1:10 (w/v) and stirred for 30 min and then ultracentrifuged at 30,000 g for 15 min at 4°C. The floated phrase was collected and the non-rubber fractions were discarded. The crude C-serum was directly ultracentrifuged at 30,000 g for 15 min at 4°C and the upper clear phase was collected as purified C-serum. To purify the lutoids, the collected crude lutoids were added into the ice-cold WS in ration of 1:10 (w/v) as above and incubated for 10 min on ice, and then ultracentrifuged at 30,000 g for 15 min with 4°C. The bottom pellet was collected as purified lutoids.

### Total protein extraction protocol for different rubber latex fractions

This protocol was developed from our recently reported BPP method [20,21], thus called the modified BPP protocol. The fresh total latex and different fractions for rubber particles, C-serum and lutoids of rubber latex were respectively homogenized in the BPP buffer (100 mM EDTA, 100 mM Tris (pH 8.0), 50 mM Borax, 50 mM Vitamin C, 1% PVPP (w/v), 1% Triton X-100 (v/v), 2% β-mercaptoethanol (v/v) and 30% sucrose (w/v)) in ration of 1:5 (v/v for C-serum; w/v for rubber particle and lutoid). Then, these mixtures were vortexed vigorously for 30 min at room temperature. For total latex, the mixture was sonicated for 5 min at 70 W/cm^2 ^on ice with an ultrasonic processor UP200 S, then centrifuged at 15,000 g for 15 min with 4°C, and the lower clear phase was collected as purified total latex. For rubber particle, the mixture was sonicated as above and then centrifuged at 15,000 g for 15 min with 4°C, and the lower clear phase was collected as purified rubber particle fraction. Subsequently, 2 volumes of Tris-saturated phenol (pH 8.0) were added into the purified fractions of total latex, rubber particles, C-serum and lutoids respectively and then the mixtures were further vortexed for 15 min at room temperature.

After centrifugation (4°C, 15 min, 15 000 *g*), the upper phase was transferred into a new centrifuge tube. Equal volume of extraction buffer was added into the new tube, the mixture was then vortexed for 10 min, followed by centrifugation at the same condition. The upper phase was then transferred into a new centrifuge tube. Proteins were precipitated by adding 5 volumes of ammonium sulfate saturated-methanol, and incubating at -20°C for at least 6 hours. After centrifugation as above, the protein pellet was resuspended and rinsed with ice-cold methanol followed by ice-cold acetone twice, and spun down at 15 000 *g *for 5 min at 4°C after each washing, and then the mixture was carefully decanted. Finally, the washed pellet was air-dried, and recovered with Lysis buffer (7 M urea, 2 M thiourea, 2% CHAPS, 13 mM DTT, 1% IPG buffer).

### Membrane protein extraction from lutoids

The purified lutoids were resuspended in 3 volumes of WS and submitted to repeated freezing and thawing 3 times (at -20°C and 37°C) to rupture the lutoid membranes and thus released their fluid content, followed by centrifugation at 15,000 g for 15 min at 4°C as described [8]. The bottom fraction was then resupended in 10 volumes of WS and performed sucrose density-gradient centrifugation to obtain the purified organelles as described [25]. The collected pellets were stirred with equal volume of WS and sonicated for 5 min at 70 W/cm^2 ^on ice, then centrifugated at 15,000 g for 15 min at 4°C to collect the pellets. The collected bottom pellets were resuspended in 10 volumes of BPP buffer to extract the lutoid membrane proteins as same as the above protocol for lutoids.

### Protein quantification

Protein concentration was determined according to the Bradford method by the spectrophotometer (Shimadzu UV-160, Kyoto, Japan). BSA was used as the standard.

### 1-DE and 2-DE

1-DE was carried out in a 16 cm slab gel apparatus using 12.5% polyacrylamide as the separating gel and 4% polyacrylamide as the stacking gel. About 30 μg proteins were loaded per lane for 1-DE. About 800 μg proteins was loaded onto an IPG strip holder with the 24 cm linear IPG strips of pH 4-7, and rehydrated for 24 h at room temperature. Then the strips were subject to IEF in an Ettan IPGphor system according to the manufacturer's instruction (2-DE Manual, GE Healthcare).

### Gel staining and image analysis

The gels were visualized by our modified CBB R250 staining protocol [26] and scanned at 600 dots per inch, then analyzed with Image Master 2 D Platinum Software (Version 5.0, GE Healthcare).

### Protein identification *via *mass spectrometry

The proteins were identified by MALDI TOF/TOF MS/MS as described [20,22]. Firstly, the proteins were digested in-gel with bovine trypsin as described [27]. After digestion, the peptides were collected and vacuum dried for MS. Mass spectra were obtained on an Autoflex MALDI TOF/TOF mass spectrometer instrument with TOF-TOF facility and MALDI with MS/MS (Bruker Daltonics, Billerica, MA, USA) equipped with a pulsed N_2 _laser (337 nm). Then the spectra were analyzed with flexAnalysis software (Version 3.2, Bruker-Daltonics, USA) and search against the taxonomy of Viridiplantae (Green Plants) in the nonredundant NCBI (NCBInr) database using MASCOT software (Version 2.2). The PMF searched parameters were: 300 ppm tolerance as the maximum mass error, MH^+ ^monoisotopic mass values, allowance of oxidation (M) modifications, allowed for 1 missed cleavage, and fixed modification of cysteine by carboxymethyl (Carbamidomethylation, C). In addition, an MS/MS ion search was done under the above searched conditions except for an MS/MS tolerance of ± 0.3 Da. MALDI-TOF/TOF fragment ion analysis was carried out in the LIFT mode of the instrument. Then, in order to further confirm the identification, all MS/MS data from LIFT TOF/TOF spectra were combined with the corresponding MS PMF data for database searching. Since the *H. brasiliensis *genome sequences are not known, a homology based search was performed. Wherein the peptides were matched to multiple members of a protein family, or a protein appeared under different names and accession numbers, the match was considered in terms of a higher Mascot score with both TOF and TOF/TOF results, the putative functions, and differential expression patterns on 2-DE gels. Good matches were classified as those having a Mascot score higher than 71 (threshold). The identification was considered only with a higher Mascot score, maximum peptide coverage and additional experimental confirmation of the protein spots on the 2-DE gels. To avoid false positives, an additional in-house BLAST search at NCBI http://blast.ncbi.nlm.nih.gov/Blast.cgi was done to confirm all the matches.

### Statistical analysis

The statistical results were presented as means ± SE (standard error). Statistical analysis, one-way ANOVA, and Duncan's multiple range tests were performed with 5% level of significant using the SPSS software (version 12.0).

## Abbreviations

BSA: bovine serum albumin; CBB: Coomassie Brilliant Blue; CHAPS, 3-(3-cholamidopropyl)-dimethylammonio-1-propanesulfonate; DTT, dithiothreitol; EDTA, ethylene diaminetetraacetic acid; IEF: isoelectric focusing; IPG: immobilized pH gradient; MALDI-TOF MS: matrix-assisted laser desorption ionization time-of-flight mass spectrometry; *p*I: isoelectric point; PMF: peptide mass fingerprinting; PMSF: phenylmethylsulfonyl fluoride; PVPP: polyvinyipoiypyrrolidone; SDS-PAGE: sodium dodecyl sulfonate polyacrylamide gel electrophoresis; TCA: trichloroacetic acid; Tris: tris(hydroxymethyl) aminomethane; 2-DE: two-dimensional gel electrophoresis.

## Competing interests

The authors declare that they have no competing interests.

## Authors' contributions

Xuchu Wang and Weimin Tian designed and executed all experiments, performed the data analysis and wrote the manuscript. Anping Guo and Ming Peng were scientific leaders and responsible for the experimental design, supervision and revising of the manuscript. Minjing Shi and Xiuli Lu performed 2-DE, image analysis, and participated in the writing of the manuscript. Ruifeng Ma and Chenggong Wu conducted sample preparation and protein identification. All authors have read and approved the final manuscript.
